# COVID-19 Vaccine Acceptance and Uptake in Bangkok, Thailand: Cross-sectional Online Survey

**DOI:** 10.2196/40186

**Published:** 2023-04-13

**Authors:** Christopher Remmel, Gaurav Tuli, Tanner J Varrelman, Aimee R Han, Pakkanan Angkab, Hathairat Kosiyaporn, Chanikarn Netrpukdee, Supatnuj Sorndamrih, Thaksaphon Thamarangsi, John S Brownstein, Christina M Astley

**Affiliations:** 1 Computational Epidemiology Lab Boston Children’s Hospital Boston, MA United States; 2 International Health Policy Program Ministry of Public Health Nonthaburi Thailand; 3 ThaiHealth Promotion Foundation Bangkok Thailand; 4 Faculty of Medicine Harvard Medical School Cambridge, MA United States; 5 Division of Endocrinology Boston Children’s Hospital Boston, MA United States; 6 Broad Institute of Harvard and MIT Cambridge, MA United States

**Keywords:** COVID-19 vaccines, Thailand, survey, vaccines, COVID-19, pandemic, public health, health policy, epidemiology, social media, vaccine hesitancy

## Abstract

**Background:**

The third most severe COVID-19 wave in the middle of 2021 coincided with the dual challenges of limited vaccine supply and lagging acceptance in Bangkok, Thailand. Understanding of persistent vaccine hesitancy during the “608” campaign to vaccinate those aged over 60 years and 8 medical risk groups was needed. On-the-ground surveys place further demands on resources and are scale limited. We leveraged the University of Maryland COVID-19 Trends and Impact Survey (UMD-CTIS), a digital health survey conducted among daily Facebook user samples, to fill this need and inform regional vaccine rollout policy.

**Objective:**

The aims of this study were to characterize COVID-19 vaccine hesitancy, frequent reasons for hesitancy, mitigating risk behaviors, and the most trusted sources of COVID-19 information through which to combat vaccine hesitancy in Bangkok, Thailand during the 608 vaccine campaign.

**Methods:**

We analyzed 34,423 Bangkok UMD-CTIS responses between June and October 2021, coinciding with the third COVID-19 wave. Sampling consistency and representativeness of the UMD-CTIS respondents were evaluated by comparing distributions of demographics, 608 priority groups, and vaccine uptake over time with source population data. Estimates of vaccine hesitancy in Bangkok and 608 priority groups were tracked over time. Frequently cited hesitancy reasons and trusted information sources were identified according to the 608 group and degree of hesitancy. Kendall tau was used to test statistical associations between vaccine acceptance and vaccine hesitancy.

**Results:**

The Bangkok UMD-CTIS respondents had similar demographics over weekly samples and compared to the Bangkok source population. Respondents self-reported fewer pre-existing health conditions compared to census data overall but had a similar prevalence of the important COVID-19 risk factor diabetes. UMD-CTIS vaccine uptake rose in parallel with national vaccination statistics, while vaccine hesitancy and degree of hesitancy declined (−7% hesitant per week). Concerns about vaccination side effects (2334/3883, 60.1%) and wanting to wait and see (2410/3883, 62.1%) were selected most frequently, while “not liking vaccines” (281/3883, 7.2%) and “religious objections” (52/3883, 1.3%) were selected least frequently. Greater vaccine acceptance was associated positively with wanting to “wait and see” and negatively with “don’t believe I need (the vaccine)” (Kendall tau 0.21 and −0.22, respectively; adjusted *P*<.001). Scientists and health experts were most frequently cited as trusted COVID-19 information sources (13,600/14,033, 96.9%), even among vaccine hesitant respondents.

**Conclusions:**

Our findings provide policy and health experts with evidence that vaccine hesitancy was declining over the study timeframe. Hesitancy and trust analyses among the unvaccinated support Bangkok policy measures to address vaccine safety and efficacy concerns through health experts rather than government or religious officials. Large-scale surveys enabled by existing widespread digital networks offer an insightful minimal-infrastructure resource for informing region-specific health policy needs.

## Introduction

The third COVID-19 wave in Thailand, spanning June to October 2021, presented a serious challenge to health infrastructure [1-3]. A rapid increase in COVID-19 severity and spread coincided with slow vaccine rollout to nonhealth care workers. Vaccine distribution was further impeded by limited supply and lagging demand. Thailand initially targeted vaccination of the “608” group that included high-risk people defined as those aged 60 years or older and those with 1 of 8 pre-existing health conditions (PHCs) such as diabetes, respiratory disease, or pregnancy [4,5]. The government offered expanded vaccine supply and options [6,7], and focused institutional support in conjunction with incentives and, in some instances, punitive measures [8-10]. Nevertheless, vaccine hesitancy remained a persistent public health problem in Thailand [11], and yet, studies of vaccine hesitancy trends in the region were sparse. There was an urgent need to understand these trends, understand the reasons for vaccine hesitancy, and understand how best to persuade the unvaccinated in Thailand.

Vaccine hesitancy literature on the psychological and sociological drivers of this public health issue identified common correlates such as lack of social pressure, complacency toward the disease, lack of trust in medicine, false beliefs about vaccination, lack of trust in the government, and conspiratorial thinking [12]. Specific reasons for vaccine hesitancy in Thailand were often connected to fears of side effects and of lack of benefits, most notably regarding the Sinovac vaccine offered by the government early in vaccine rollout [13,14]. However, these surveys were either narrowly focused on particular subgroups, such as health care workers or seniors, or were otherwise offered for short windows of time [13,15-20]. Policy and public health officials working to change sentiment and behaviors in the region might use data from more detailed longitudinal studies inclusive of broader population demographics.

The University of Maryland Global COVID Trends and Impact Survey (UMD-CTIS) is an innovative data stream covering the health attitudes and practices of a large population sample (the global Facebook active user base [FAUB]) from early in the COVID-19 pandemic (April 2020). A large proportion of the population of Thailand, especially Bangkok, is represented in the FAUB. As such, the UMD-CTIS was well suited to address the limitations of prior surveys and fill the need for context-specific information to combat vaccine hesitancy in the region. Leveraging UMD-CTIS responses from the residents of Bangkok, the most populous and most densely populated urban area with the highest COVID-19 burden [21], we sought to understand vaccine uptake, attitudes, and opportunities to better address vaccine messaging in the 608 vaccine rollout era.

## Methods

### Study Design

#### UMD-CTIS

This research is based on survey responses from the UMD-CTIS, which has been approved by the University of Maryland (UMD) Institutional Review Board (1587016-10) and has been described previously [22]. Briefly, the UMD-CTIS, in partnership with Facebook, is a cross-sectional survey of daily samples of the FAUB population. Sampled FAUB users were invited to participate in the UMD-CTIS through a special banner. Respondents aged ≥18 years who consented to participate in the UMD-CTIS study completed an online Qualtrics survey administered by the UMD [23]. Though statistical resampling of users from the FAUB over time is possible, these resampled FAUB users cannot be identified, and their UMD-CTIS responses cannot be linked longitudinally by design.

#### Survey Instrument

The UMD-CTIS instrument was designed to evaluate a range of public health topics through a single cross-sectional survey requiring limited time burden. Survey questions were updated by the UMD periodically in response to evolving epidemiologic needs. A consistent survey instrument (version 11) was used during the study period described below. This instrument covered vaccination status and vaccine hesitancy, in addition to questions on demographics, knowledge, attitudes, health practices, and health status. One of two additional survey modules was offered randomly to each respondent, either module A (media trust) or module B (PHCs). Survey logic and language (English and Thai) for version 11 are summarized in Multimedia Appendix 1, and are available online [24].

Because survey questions administered to each respondent target different subject matter by design and responses from the resampled FAUB cannot be linked, possible dependence of observations and measures of internal consistency are not available. Assuming that the large FAUB population, statistical sampling scheme, and response/participation rates are stable relative to the study timescale, respondents are a consistent sample of the FAUB population. Respondent characteristics (eg, age and gender) may be used as a measure of sampling response consistency for those characteristics. Trends in survey-estimated metrics (eg, vaccination proportion among UMD-CTIS respondents) are assumed to be estimates of trends in the source population for the FAUB population. The UMD-CTIS compared to external benchmark data may be used as a measure of the representativeness of FAUB respondents relative to the source population of interest for those survey questions.

#### UMD-CTIS Study Population in Bangkok, Thailand

This study analyzed UMD-CTIS responses from self-identified residents of Bangkok, Thailand from June 14, 2021, to October 4, 2021. This period coincided with the third COVID-19 wave and the 608 campaign rollout. To evaluate for possible differences among those targeted in the 608 campaign, we separately analyzed 2 subgroups of survey responses representative of those groups, that is, respondents self-reporting older age and self-reporting at least one of the targeted PHCs.

The UMD-CTIS in conjunction with Facebook provided survey weights, which combine design weights (for disproportional population stratification sampling), nonresponse weights (inverse of response propensity), and poststratification weights (standardizing to regional age-gender distributions), to simultaneously adjust for regional demographics, nonresponse bias, and sampling bias, as described previously in greater detail [23]. The individual component weights are not provided by design. Thus, when evaluating subregional data (eg, PHC subgroups), it is not possible to apply only the design and nonresponse weights, without also applying poststratification weights for the region. Estimates with and without survey weights were nevertheless similar both regionally and in subgroups, and are presented for comparison in Multimedia Appendices 2-4.

### Covariates and Outcome Measures

#### Demographics and 608 Campaign Groups

Respondents self-reported demographic characteristics, including 4 gender and 7 age categories. UMD-CTIS age categories were in 10-year brackets, which did not align with the 60-year-old threshold in the 608 campaign. To avoid including subjects aged 55 to 59 years, the elderly risk group was identified as those aged ≥65 years (ie, selected 65-74 or ≥75 years UMD-CTIS age categories). When evaluating age-gender distributions, gender was limited to binary responses (“male” and “female”). Those with self-reported targeted PHCs in the 608 campaign were identified from the subpopulation of respondents randomly offered survey module B. Those who received module B did not receive module A. We included surveys from those who received module B and reported at least one of the targeted PHCs on module B. Pregnancy was only queried among those not reporting male gender. The 608 campaign PHCs were mapped to reasonable UMD-CTIS proxies of those PHCs. These included pregnancy, diabetes, obesity, cancer, chronic kidney disease, chronic respiratory diseases (asthma and chronic obstructive pulmonary disease combined), and cardiovascular diseases (heart attack, heart disease, or other heart conditions). The 608 campaign PHCs of neurovascular diseases did not have reasonable proxies in the UMD-CTIS and were not evaluated.

#### Vaccine Uptake

We evaluated vaccine uptake in the UMD-CTIS in Bangkok and the 608 subpopulations relative to the publicly reported vaccine uptake measures. Self-reported vaccination status was ascertained using the questions “Have you had a COVID-19 vaccination?” and “How many COVID-19 vaccinations have you received?” Complete vaccination was assessed with the response “Vaccinated, two doses,” which is consistent with the primary formulations available widely during the study period. Additionally, we evaluated uptake trends by assessing the responses “Vaccinated, one dose” and “Scheduled” (for vaccination) to the question “Do you have an appointment to receive a COVID-19 vaccine?” Vaccine uptake in the region was trended over the course of the study period.

#### Vaccine Acceptance and Hesitancy

The degree of vaccine acceptance was characterized as a nominal scale with decreasing willingness to be vaccinated. The 3 groups with the highest vaccine acceptance were those fully vaccinated (“Vaccinated, two doses”), partially vaccinated (“Vaccinated, one dose”), and about to be vaccinated (“Scheduled”). The remaining unvaccinated and unscheduled respondents were categorized into 4 groups of increasing hesitancy to become vaccinated according to their responses to the question “If a vaccine to prevent COVID-19 was offered to you today, would you choose to get vaccinated?” The 4 groups were as follows: “Definitely,” “Probably,” “Probably not,” and “Definitely not.” Multimedia Appendix 1 details the survey questions, response options, and survey logic for the following questions: “Have you had a COVID-19 vaccination?” “How many COVID-19 vaccinations have you received?” “Do you have an appointment to receive a COVID-19 vaccine?” and “If a vaccine to prevent COVID-19 was offered to you today, would you choose to get vaccinated?” Surveys with missing responses to enable categorization into these 7 groups were excluded.

#### Reasons for Vaccine Hesitancy

Reasons for vaccine hesitancy were examined among the 4 most vaccine hesitant subgroups (“Definitely,” “Probably,” “Probably not,” and “Definitely not”). The question stem text varied slightly to align with the respondents’ self-identified degree of willingness to be vaccinated (ie, “Which of the following, if any, are reasons that you definitely wouldn’t/probably wouldn’t/only probably would choose to get a COVID-19 vaccine?”). Hesitancy reasons were presented as multichoice responses and included a range of options from concerns about vaccine side effects to beliefs about vaccine efficacy (Multimedia Appendix 1).

#### Reasons for Not Believing Vaccination is Necessary

Hesitant respondents who endorsed “I don’t believe I need a vaccine” were further questioned for their reasons for holding that belief. Proportions were calculated for each individual reason and for the incidence of co-selected pairs in this multichoice response.

#### Trusted Media for COVID-19 Information

Potential avenues for communicating with vaccine hesitant individuals in the region were evaluated among the 4 most vaccine hesitant subgroups and those who accepted vaccination in some form (scheduled or received any number of doses). Survey module A surveyed preferred sources of information relating to COVID-19. This module was presented to a random subset of respondents; those who received module A did not receive module B. Subjects were asked “How much do you trust the following sources to provide accurate news and information about COVID-19?” The 3 response options “Trust,” “Somewhat trust,” and “Do not trust” were dichotomized by combining the first two into a single “Trust” option. For each respondent, “trusted sources” are therefore sources of COVID-19 information that respondents indicated they “Somewhat trust” or “Trust.”

### External Data and Benchmarking

To compare the Bangkok UMD-CTIS estimates and trends to external benchmark measures of population-level statistics, we compiled population demographics, PHCs, and vaccine data from publicly available sources. Further methodology, data sources, and results are provided in Multimedia Appendix 5. To compare the Bangkok UMD-CTIS vaccine uptake relative to government-reported trends over the 608 campaign study period, we estimated the proportion of fully vaccinated (2-dose) respondents in the UMD-CTIS over time. The daily sample size of the Bangkok UMD-CTIS is small in comparison to the rate of change in the source population vaccinated proportion. Thus, to show temporal trends in vaccine uptake in the Bangkok UMD-CTIS study over time, 28-day rolling averages of the proportion vaccinated are shown in relation to the national daily reports. Bangkok vaccination data over time were obtained from the Thailand Department of Disease Control daily reports [25]. Complete vaccination per population was calculated using data on 2 doses administered as described above, consistent with the daily vaccination reports.

### Statistical Analyses

Estimates and CIs for proportions were calculated with the Wilson Score method, using the count of affirmative responses per total survey in the subgroup of interest, unless noted otherwise. For multichoice questions (eg, PHCs and hesitancy reasons), we used the count for that choice item. Survey-weighted and raw proportions are shown in Multimedia Appendix 5, though subgroup demographics differed from Bangkok, Thailand demographics from which UMD-CTIS weights were, in part, derived. A test of the trend for demographic differences between the UMD-CTIS and census over time was conducted using linear regression and applying Bonferroni correction for multiple comparisons to generate an adjusted *P* value. Among hesitant respondents, we evaluated associations between the degree of vaccine acceptance and reasons for hesitancy by using Kendall tau and applying Bonferroni correction for multiple comparisons. We repeated this analysis for 608 campaign group subsets.

### Ethics Approval

The Institutional Review Board of Boston Children’s Hospital (P00023700) approved this study that used the UMD-CTIS.

## Results

### Bangkok UMD-CTIS

#### Demographics of the Bangkok UMD-CTIS

Table 1 shows that the Bangkok UMD-CTIS respondents (N=36,334) were more frequently male and under 65 years of age. There were 1265 (3.5%) respondents in the ≥65 years elderly risk group targeted by the 608 campaign. Of the 19,734 respondents who received the PHC survey module, 3407 (17.3%) reported having at least one of the 7 PHCs covered by the survey. Diabetes (1090/19,734, 5.5%) and obesity (1555/19,734, 7.9%) were the most frequently reported PHCs targeted in the 608 campaign. Comparisons of the Bangkok UMD-CTIS and 608 subgroups to benchmark census data, along with survey weight-adjusted estimates, are presented in Multimedia Appendix 5. Multimedia Appendix 6 shows that demographics were consistent over time, while in Multimedia Appendix 7, linear regression results for demographic differences between the UMD-CTIS and census showed that adjusted *P* values never met the threshold for significance and coefficients were always close to zero. While Bangkok UMD-CTIS respondents were similar to the Bangkok general population in terms of age, gender, and diabetes status, Bangkok UMD-CTIS respondents, who were selected from the Bangkok FAUB population, indicated that they were healthier than the general population with respect to other COVID-19 risk factors.

**Table 1:** Demographic and pre-existing health condition characteristics of survey respondents in the cross-sectional survey (June 14, 2021, to October 4, 2021).

Characteristic		Value, n (%)
**Age group (years) (N=36,334)**		
	18-24	2759 (7.6)
	25-34	7641 (21.0)
	35-44	8907 (24.5)
	45-54	6439 (17.7)
	55-64	3569 (9.8)
	65-74	1102 (3.0)
	≥75	163 (0.4)
	Not reported	5754 (15.8)
**Sex (N=36,334)**		
	Male	16,308 (44.9)
	Female	13,468 (37.1)
	Other	331 (0.9)
	Prefer not to answer	477 (1.3)
	Not reported	5750 (15.8)
**PHC^a,b^ (N=19,734)**		
	At least one condition	3407 (17.3)
	Obesity	1555 (7.9)
	Diabetes	1090 (5.5)
	Chronic respiratory diseases^c^	641 (3.2)
	Asthma	575 (2.9)
	Cardiovascular diseases	488 (2.5)
	Chronic kidney disease	207 (1.0)
	Cancer	171 (0.9)
	Chronic lung diseases	155 (0.8)
	Pregnancy^d^	92 (0.3)
**Vaccine uptake and hesitancy (N=36,334)**		
	Vaccinated, 2 doses	8596 (23.7)
	Vaccinated, 1 dose; unspecified dose	13,712 (37.7)
	Scheduled	5505 (15.2)
	Definitely willing	2909 (8.0)
	Probably willing	2619 (7.2)
	Probably not willing	652 (1.8)
	Definitely not willing	522 (1.4)

^a^PHC: pre-existing health condition.

^b^Of the 36,334 respondents, 19,734 (54.3%) received the module about PHCs and 16,600 (45.7%) did not receive the module.

^c^Includes respondents who self-identified being diagnosed with asthma or chronic lung diseases such as chronic obstructive pulmonary disease, chronic bronchitis, or emphysema.

^d^A total of 7405 (25.0%) responded “No.”

### Vaccine Uptake

**Figure 1:**
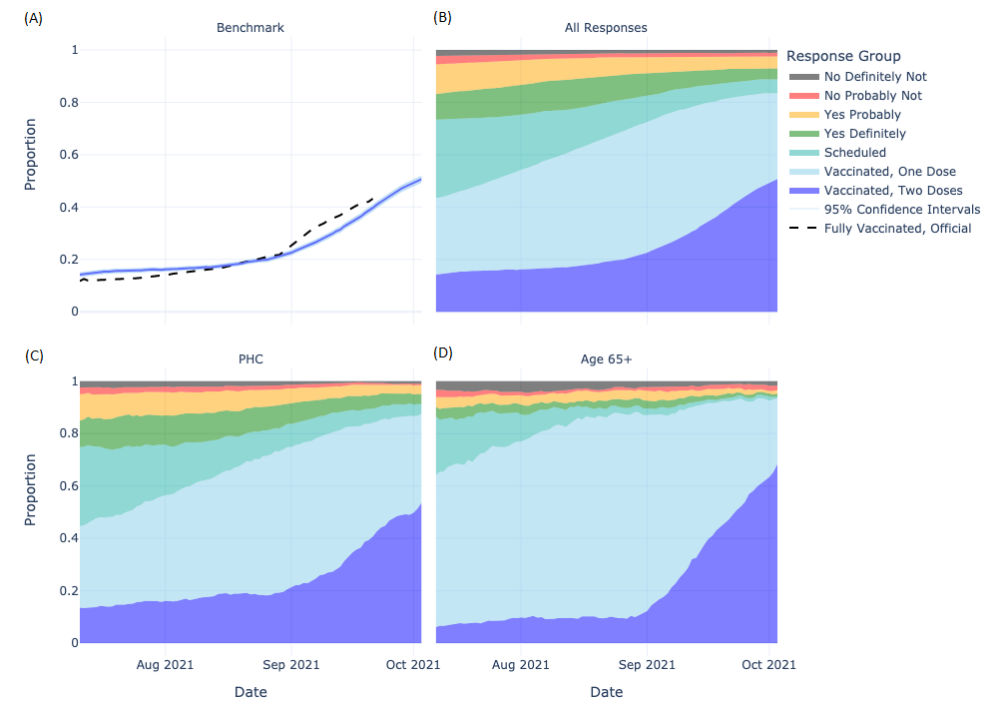
Vaccine uptake and acceptance in Bangkok over time and within 608 vaccination campaign priority groups from June 14, 2021, to October 4, 2021. (A) The 4-week moving average government vaccination uptake trend (dashed line) compared against the fully vaccinated trend in the COVID-19 Trends and Impact Survey overall (dark blue line) with 95% CIs (light blue line). (B-D) Stacked proportion of respondents indicating vaccine acceptance (2-dose vaccinated, dark blue; 1-dose vaccinated, blue; scheduled, turquoise; definitely, green; probably, yellow; probably not, red; definitely not, black) across all responses (B), and further faceted across pre-existing health condition (PHC) (C) and elderly (D) subgroups.

In Figure 1, we show vaccine uptake tracked over the 608 campaign study period using the proportion of Bangkok UMD-CTIS respondents who indicated they were scheduled to or had received one or two doses of the COVID-19 vaccine. Vaccination trends in 2-dose vaccinated respondents closely mirrored Bangkok government statistics (Figure 1A). The proportion scheduled to be vaccinated declined (−14.0%/week) as the proportion vaccinated with at least one dose rose. While elderly people received their first dose more quickly than others (158/246, 64.2% vs 3305/7617, 43.4% with at least one dose at the start of the study period), uptake of the second dose increased more gradually during the summer. Uptake patterns among those with PHCs were not noticeably different from responses overall. The 95% CIs were very small owing to the large sample size (Multimedia Appendix 8). The UMD-CTIS–weighted trends were similar. 

### Vaccine Acceptance and Hesitancy

To evaluate vaccine hesitancy over time, we classified unvaccinated and unscheduled respondents according to their degree of vaccine acceptance. The proportions of those who said that they would probably, probably not, and definitely not get vaccinated steadily declined, though more gradually (−7.3%/week) compared to the rise in vaccine uptake (Figure 1). Vaccination hesitancy did not increase over the study period overall or within the 608 targeted groups (Figure 1). However, the rise in vaccine uptake appeared to mirror changes in those who were scheduled to receive vaccination and accepting of vaccination, rather than a decline in those hesitant to do so.

### Reasons for Vaccine Hesitancy

**Figure 2:**
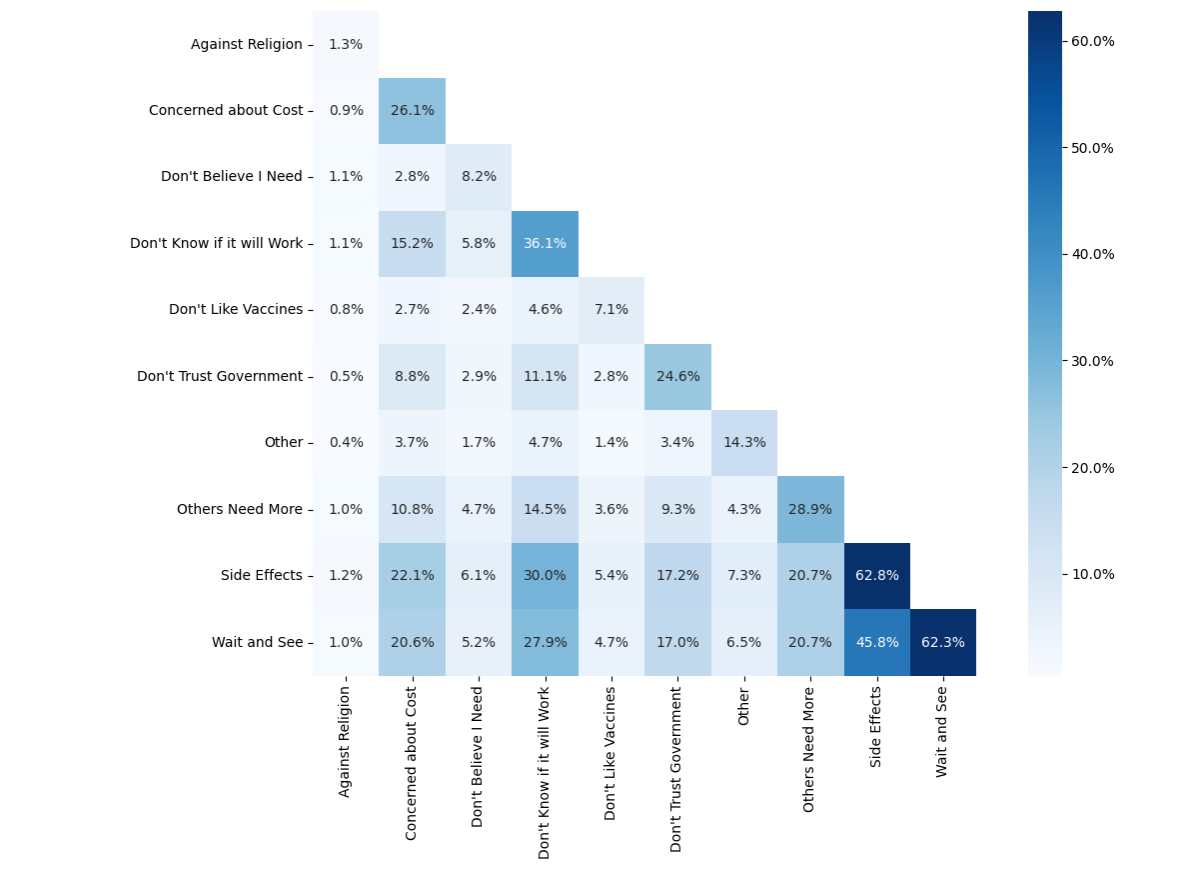
Frequency matrix of hesitancy reasons in the cross-sectional survey (June 14, 2021, to October 4, 2021). Intersection of 2 reasons is the frequency that both reasons were chosen by a respondent. Intersection of a reason with itself is the frequency for that single reason across all respondents.

We evaluated the reasons cited by the 3 groups of hesitant respondents who indicated they would probably, probably not, or definitely not get vaccinated (N=3883). Figure 2 shows that reasons relating to the risk-benefit of vaccination were more frequently selected among hesitant respondents. Respondents reported concerns over side effects (2434/3883, 62.7%), wanting to wait and see for longer to determine whether the vaccines are safe (2410/3883, 62.1%), and not feeling sure that the vaccines will be effective in protecting against COVID-19 (1407/3883, 36.2%). Concerns about side effects and lack of vaccine benefit were the most frequently selected together with wanting to wait and see (45.8% and 27.9%, respectively).

**Figure 3:**
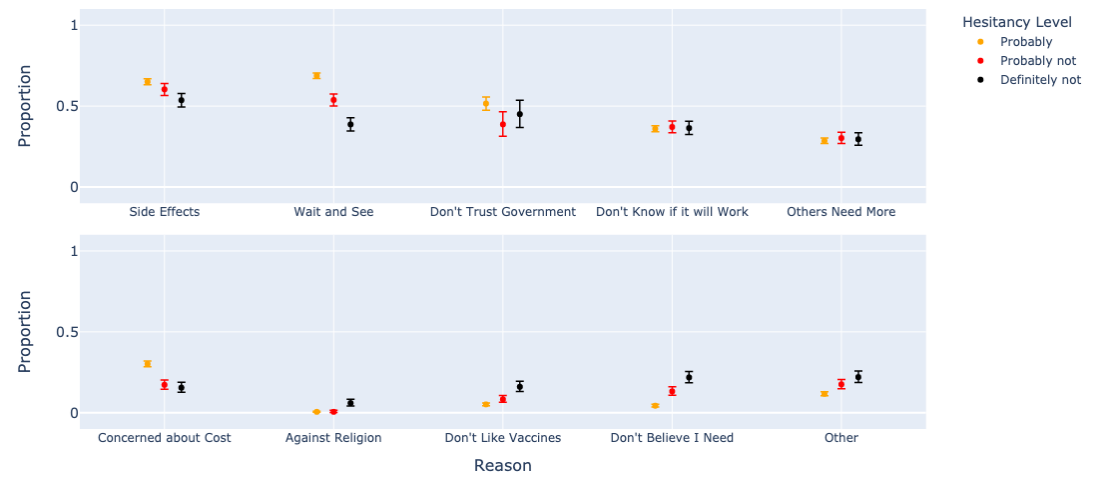
Reasons for hesitancy by degree of vaccine acceptance in the cross-sectional survey (June 14, 2021, to October 4, 2021).

Figure 3 shows that when stratifying according to respondents’ vaccine acceptance, risk-benefit concerns were similar in frequency. In Table 2, we see that the strength of the association of endorsing a specific concern with acceptance of vaccination was small, with Kendall tau mostly below ±0.2, even though adjusted *P* values were mostly <.001 from the large survey size. The most hesitant were more averse to vaccination (“Don’t believe I need,” tau=−0.22; “Don’t like vaccines,” tau=−0.13) and were less likely to consider vaccination later (“Wait and see,” tau=+0.2). The most hesitant were nevertheless unsure of the benefits of vaccination, similar to the less hesitant groups (“Don’t know if it will work,” tau=−0.01). Those remaining unvaccinated and hesitant at the end of the study period (August 27 to October 3, 2021) selected “Don’t believe I need” more frequently than at the start of the study period, while “Don’t know if it will work” did not shift over time.

In Multimedia Appendix 9, we see that among the 608 campaign groups, the reasons for hesitancy patterns were broadly similar to the patterns for all respondents. The unvaccinated and hesitant respondents aged ≥65 years selected “Don’t believe I need” more frequently but were similarly most often concerned about “side effects.”

**Table 2:** Association between vaccine acceptance and individual reasons for hesitancy measured by Kendall tau in the cross-sectional survey (June 14, 2021, to October 4, 2021).

Hesitancy reason	Kendall tau	Adjusted *P* value
Wait and see	0.21	<.001
Concerned about cost	0.14	<.001
Side effects	0.08	<.001
Don’t know if it will work	−0.01	>.99
Others need it more	−0.01	>.99
Other	−0.1	<.001
Against religion	−0.11	<.001
Don’t like vaccines	−0.13	<.001
Don’t believe I need	−0.22	<.001

### Reasons for Not Believing Vaccination is Necessary

**Figure 4:**
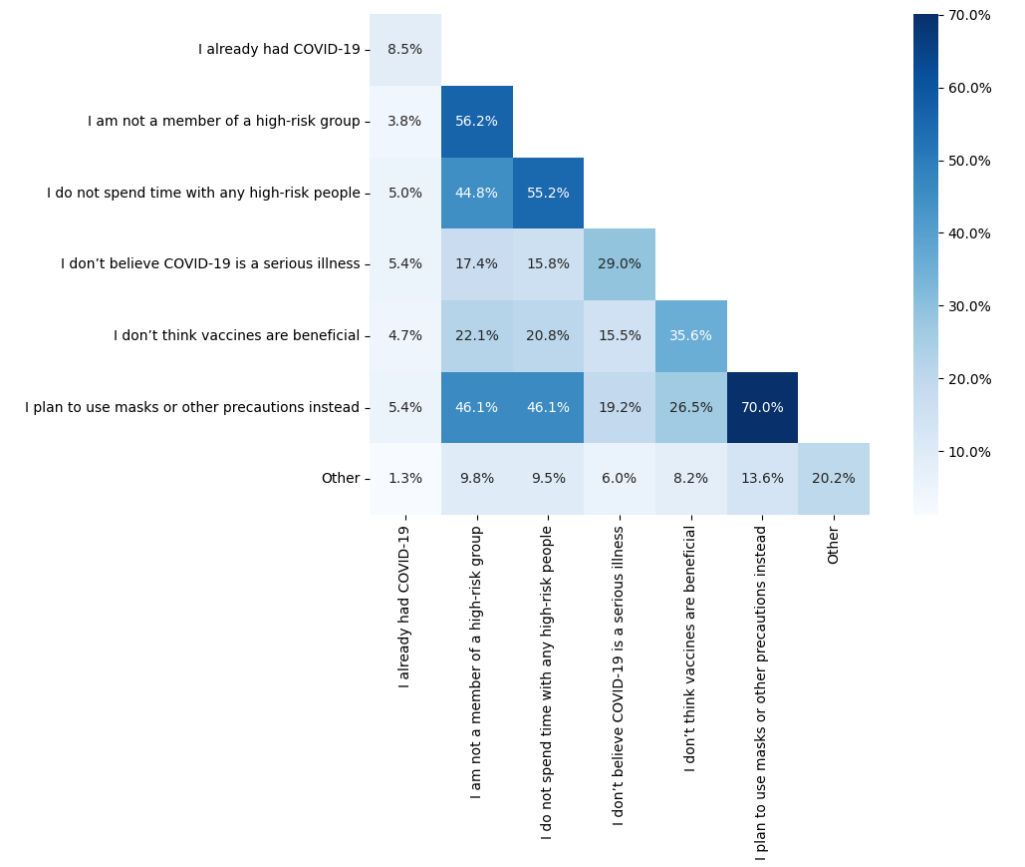
Frequency of selected multichoice responses for why individuals chose “I don’t believe I need a COVID-19 vaccine” in the cross-sectional survey (June 14, 2021, to October 4, 2021).

Given the consistent endorsement of “I don’t believe I need a COVID-19 vaccine” among the unvaccinated and most hesitant respondents across risk groups and over time, we further evaluated the behaviors and attitudes of respondents who selected this reason for hesitancy. In Figure 4, we see that those who did not believe they needed a COVID-19 vaccine (N=317) generally endorsed other protective behaviors and beliefs overall and within the 608 campaign groups. They most frequently reported their intention to mask or pursue other mitigation strategies instead of vaccination (222/317, 70.0%), and least frequently indicated low confidence in vaccines (113/317, 35.6%) or in COVID-19 as a serious illness (92/317, 29.0%).

Over half (35/60, 58%) of unvaccinated hesitant respondents who did not believe they needed a COVID-19 vaccine did not endorse being a member of a high-risk group, even though they self-reported older age or a PHC in the 608 campaign. Nevertheless, Multimedia Appendix 10 shows that most respondents who indicated they were not in a risk group also planned to use protective measures other than vaccines, and infrequently indicated they did not think COVID-19 was a serious illness. Thus, while unvaccinated hesitant people in the 608 campaign groups may not be aware of their underlying COVID-19 risk, such as older age or diabetes status, they do appear to have an appreciation of the risk and an understanding of important risk mitigation measures.

### Trusted Media for COVID-19 Information

**Figure 5:**
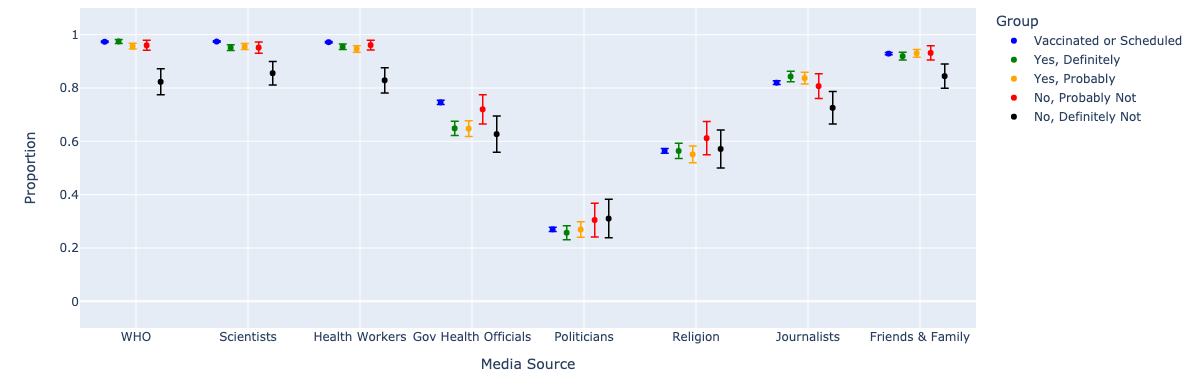
Trusted sources of information on COVID-19 by grade of vaccine hesitancy. Frequency of selected multichoice options for trusted media sources, combining responses for most trusted and somewhat trusted.

Given vaccine hesitancy concerns and respondents indicating they might vaccinate later if there is more information, we sought to investigate what information sources would best reach this group. Medical and scientific representatives were the most trusted sources of information on COVID-19 among all respondents, regardless of the degree of hesitancy. Specifically, in Figure 5, we see that respondents who completed each of these questions most frequently indicated that they trust the World Health Organization (13,564/13,980, 97.0%), scientists/health experts (13,600/14,033, 96.9%), and local health workers (13,827/14,304, 96.7%) for news relating to the pandemic. Politicians (3723/13,794, 27.0%) and religious leaders (7668/13,794, 55.6%) were least frequently selected as trusted. The most hesitant respondents were less trusting overall but still trusted scientists and other health experts more than other sources.

## Discussion

### Principal Findings

Through leveraging daily online health surveys conducted among the residents of Bangkok, Thailand, we found that concerns about the risks and benefits of COVID-19 vaccines were paramount during the third wave, but trust in scientific sources remained intact. Globally, the pandemic and response polarized many populations. In many regions, this led to distrust of scientific evidence and interventions, and eroded the relationship among people, politicians, and public health officials [26]. We showed that, in contrast to the antiscience and antivaccine sentiment identified elsewhere [27], most unvaccinated hesitant respondents in Bangkok still believed in the seriousness of COVID-19, in the benefits of mitigation measures, and in the information relayed by health officials. The high professional trust in health experts and scientists in Thailand offered a valuable support mechanism for the government to effectively manage response efforts [28,29].

In a systematic review of the factors associated with vaccine hesitancy [26], the most important predictors included low perceived risk of infection or sequelae, low trust in institutions, low rates of influenza vaccination, and concerns regarding vaccine side effects. Prior surveys in Thailand identified unique concerns about the efficacy of vaccines from specific manufacturers, especially Sinovac [14,19]. Our findings of risk-benefit concerns among the unvaccinated echo these themes. Additionally, we identified that many hesitant respondents were planning to wait for additional information on vaccine safety and that the specific distrust of all vaccines is low (10% vs <20% in the United States [30]). Thus, messaging from health experts emphasizing vaccine safety and efficacy, and updates as more evidence on Thai-specific vaccine options becomes available, would be most effective in reaching the most hesitant unvaccinated respondents in our survey.

Among respondents within the ≥65 years and PHC risk groups, vaccine hesitancy and reasons for hesitancy were broadly similar to the general Bangkok population. Over half (35/60, 58%) of the unvaccinated respondents who provided reasons for not believing that they needed to be vaccinated and who self-reported older age and PHCs, however, did not self-identify as being at risk for COVID-19. While older age, pregnancy, and diabetes may be well-understood by a patient, other diagnoses may be less so. The burden of disease estimates for conditions, such as renal disease [31], may depend on access to testing (eg, biopsy) or may only be understood by the patient as a medical condition or risk factor when especially severe (eg, requiring medication or dialysis). This highlights the importance of 608 campaign messaging to educate citizens about the top priority vaccination groups, with the support of community health workers.

Being able to understand the unique needs of Bangkok in the context of the 608 campaign and third COVID-19 wave underscores the value of using insights from digital health surveys, such as the UMD-CTIS, to understand interventions, inform policy, and lessen the pandemic impact. Responses to health surveys may not be generalizable due to selection and response bias, and this may be compounded by sampling from the FAUB of social media users [32]. However, most Thai people have Facebook social media accounts [33], and we showed that the demographics of UMD-CTIS respondents aligned with the demographics of the Bangkok population. Thus, while there may be some bias toward respondents with more health access, awareness, or interest, the efficacy of daily sampling of the FAUB and the consistent survey instrument combined with the minimal need for on-the-ground infrastructure make this data stream a powerful resource. Additionally, observed slower uptake during the summer lines up with the time period when a longer gap between doses was recommended [34], which demonstrates the survey’s capability to identify and measure the effects of health policy changes. This analysis provides region-specific supporting evidence of resilience and adaptability in the face of a rapidly evolving public health threat and serves as a helpful validation of government efforts with support from public health experts.

### Limitations

The analyses presented here have several potential limitations. The global UMD-CTIS instrument was developed during the pandemic to measure the most critical epidemiologic measures, knowledge, attitudes, and practices in near real time. The instrument is limited by a lack of internal and external validation metrics and is thus susceptible to measurement bias [35]. Redundant questions were not included to conduct within-survey internal consistency measures. Repeat respondents could not be paired by design, and as such, the independence of observations cannot be guaranteed, which could make estimates of standard error overconfident. We found that the UMD-CTIS was consistent with 3 external benchmarks, and prior work validated other UMD-CTIS measures [36,37]. As discussed above, PHCs other than diabetes were likely undermeasured by the instrument or underrepresented in the survey sample. However, the notable variation in differences from population estimates across the PHCs suggests that other factors may also be contributing to underestimation, for example, patient understanding of the nuances of chronic kidney disease stages relative to that of the diagnosis of diabetes, which might also affect a subject’s vaccine acceptance. While digital health surveys sampled from a social media platform user base may not be generalizable to all Bangkok residents, this limitation also applies to other previously used survey methods conducted in the region to understand vaccine hesitancy, such as telephone and door-to-door surveys [17]. There is possible selection bias as subgroups that are less comfortable with social media and consumer technology use, such as very elderly people, may be underrepresented, which would mean less coverage of especially vulnerable groups. In contrast, marginalized populations, such as migrant workers affected by the case surges in the third wave [38], may be fearful of in-person interviews, but may be more comfortable responding to an anonymous survey through social media. A cross-sectional survey, such as the UMD-CTIS, may be subject to ecological bias, especially if the source population changes over time or there is confounding by time of the response pairs. Because participation in public health surveys is voluntary, there may be response bias if respondents have more of an interest in public health than the population at large, and as such, attitudes may be shifted away from hesitancy or lack of interest in mitigation. We observed relatively stable trends in respondent demographics and hesitancy attitudes over time, even with continued uptake of vaccination. The sampling scheme of the UMD-CTIS, being nested within an existing social media user base, may be less susceptible to this if the membership in the FAUB and response rate are stable over the time period under investigation.

### Conclusions

We showed that vaccine hesitancy in Bangkok during the third wave was connected to an interest in more health expert information about vaccine efficacy and safety. The UMD-CTIS findings were concordant with smaller studies highlighting skepticism about specific vaccine options available during rollout. Importantly, hesitancy among unvaccinated respondents in the UMD-CTIS was not associated with a broader distrust in science or the public health system. This may be a valuable resource for future studies to understand the regional landscapes of vaccine hesitancy in the context of specific policy interventions such as the 608 campaign in Thailand.

## Appendix

Multimedia Appendix 1 Summary of survey questions, answers, and logic for variables used in this study.

Multimedia Appendix 2 Unweighted vs weighted vaccine uptake and acceptance in Bangkok over time. Four-week moving average for the stacked proportion of respondents indicating grade of vaccine uptake and acceptance from fully vaccinated (dark blue) to definitely would not vaccinate (black). Government vaccination uptake trend (dashed line) is also visible in the fully vaccinated trend overall and further faceted across unweighted and weighted data.

Multimedia Appendix 3 Unweighted vs weighted hesitancy reasons among all respondents. Unweighted (circle) and weighted (open circle) frequencies of selected responses for vaccine hesitancy of multichoice items overall.

Multimedia Appendix 4 Unweighted vs weighted trusted sources of information on COVID-19 by grade of vaccine hesitancy. Unweighted (circle) vs weighted (line) frequencies of selected multichoice options for trusted media sources, combining responses for most trusted and somewhat trusted.

Multimedia Appendix 5 Demographic and pre-existing health condition characteristics of survey respondents and that of the general population in Bangkok.

Multimedia Appendix 6 Demographic differences by week between census and University of Maryland COVID-19 Trends and Impact Survey data.

Multimedia Appendix 7 Results for simple linear regression for week versus difference in the proportion of each of the 14 age-gender groups in University of Maryland COVID-19 Trends and Impact Survey versus Bangkok census demographics.

Multimedia Appendix 8 Vaccine uptake by group with CIs. Four-week moving average for the proportion of respondents faceted by the grade of vaccine uptake and acceptance from fully vaccinated to definitely would not vaccinate. Grey lines denote the 95% CI.

Multimedia Appendix 9 Reasons for hesitancy by grade of vaccine uptake and acceptance, and by the 608 vaccination campaign priority groups. Frequency of selected responses for vaccine hesitancy of multichoice items overall (circle) and within pre-existing health conditions (open diamond) or elderly people (open square), as well as within subgroups defined by degree of hesitancy (probably, orange; probably not, red; definitely not, black).

Multimedia Appendix 10 Reasons for not needing vaccination in the 608 vaccination campaign priority groups. Frequency of selected multichoice responses for why individuals chose “I don't believe I need a COVID-19 vaccine” as a hesitancy reason. Nonexclusively grouped by all responses (circle), pre-existing health condition (open diamond), and age 65+ years (open square).

## Abbreviations

FAUB: Facebook active user base
PHC: pre-existing health condition
UMD: University of Maryland
UMD-CTIS: University of Maryland COVID-19 Trends and Impact Survey
