# Supplementary material for: COVID-19 Vaccine Acceptance and Uptake in Bangkok, Thailand: Cross-sectional Online Survey
Source: JMIR Public Health Surveill. 2023 Apr 13;9:e40186. doi: 10.2196/40186 (PMC10141306; doi:10.2196/40186)
Supplement: Multimedia Appendix 1 [file publichealth_v9i1e40186_app1.docx]

**Multimedia Appendix 1.** Summary of survey questions, answers, and logic for variables used in this study.

| **Question text / description** | **Question text / description (Thai)** | **Responses(recode value)** | **Display Logic** | **Note** |
| --- | --- | --- | --- | --- |
| **GENERAL** |  |  |  |  |
| weight: Survey weight to adjust from FB user population to the general population |  | number(float) |  |  |
| RecordedDate: Date that the response was recorded. |  | date/time (Mountain Standard Time) |  |  |
| module: Module randomization (added in version11, not applicable if module randomization is not applied) |  | A=module A  B=module B  -99=Not applicable |  |  |
| **VACCINE** |  |  |  |  |
| V1: Have you had a COVID-19 vaccination? | ไม่มีในที่กล่าวมา | 1 = Yes  2 = No 3 = I don't know -99 = missing/valid skipped/invalid -77 = seen but unanswered | ASK ALL |  |
| V2: How many COVID-19 vaccinations have you received? | คุณฉีดวัคซีนโควิด-19 ไปแล้วกี่ครั้ง | 1 = 1 vaccination or dose  2 = 2 vaccinations or doses 3 = I don't know -99 = missing/valid skipped/invalid -77 = seen but unanswered | ASK IF V1 == 1 |  |
| V15a: Do you have an appointment to receive a COVID-19 vaccine? | คุณมีการนัดหมายเพื่อฉีดวัคซีนโควิด-19 หรือไม่ | 1 = Yes  2 = No -99 = missing/valid skipped/invalid -77 = seen but unanswered | ASK IF V1 != 1 |  |
| V3a: If a vaccine to prevent COVID-19 were offered to you today, would you choose to get vaccinated? | หากมีวัคซีนป้องกันโควิด-19 มาเสนอให้คุณในวันนี้ คุณจะเลือกฉีดวัคซีนหรือไม่ | 1 = Yes, definitely  2 = Yes, probably  3 = No, probably not  4 = No, definitely not -99 = missing/valid skipped/invalid -77 = seen but unanswered | ASK IF V1 != 1 AND V15a != 1 |  |
| V5(a/b/c): Which of the following, if any, are reasons that you (definitely wouldn't/probably wouldn't/only probably would) choose to get a COVID-19 vaccine? Please select all that apply. | ข้อใดต่อไปนี้คือเหตุผลที่ทำให้คุณไม่เลือกเข้ารับการฉีดวัคซีนโควิด-19 อย่างแน่นอน หากมี โปรดเลือกทุกข้อที่ใช่  ข้อใดต่อไปนี้คือเหตุผลที่จะทำให้คุณอาจไม่เลือกเข้ารับการฉีดวัคซีนโควิด-19 หากมี โปรดเลือกทุกข้อที่ใช่  ข้อใดต่อไปนี้คือเหตุผลที่จะทำให้คุณอาจเลือกเข้ารับการฉีดวัคซีนโควิด-19 หากมี โปรดเลือกทุกข้อที่ใช่ | 1=Selected  0=Not selected -99 = missing/valid skipped/invalid -77 = seen but unanswered | ASK IF V3a == 2 OR 3 OR 4 |  |
| V5(a/b/c)_1: I am concerned about possible side effects of a COVID-19 vaccine | ฉันกังวลเกี่ยวกับผลข้างเคียงที่อาจเกิดขึ้นจากวัคซีนโควิด-19 |  |  |  |
| V5(a/b/c)_2: I don't know if a COVID-19 vaccine will work | ฉันไม่รู้ว่าวัคซีนโควิด-19 จะได้ผลหรือไม่ |  |  |  |
| V5(a/b/c)_3: I don't believe I need a COVID-19 vaccine | ฉันไม่เชื่อว่าวัคซีนโควิด-19 จำเป็นสำหรับฉัน |  |  |  |
| V5(a/b/c)_4: I don't like vaccines | ฉันไม่ชอบวัคซีน |  |  |  |
| V5(a/b/c)_5: I plan to wait and see if it is safe and may get it later | ฉันวางแผนที่จะรอดูก่อนว่าวัคซีนนี้ปลอดภัยหรือไม่ และอาจจะฉีดวัคซีนในภายหลัง |  |  |  |
| V5(a/b/c)_6: I think other people need it more than I do right now | ฉันคิดว่าตอนนี้คนอื่นต้องการวัคซีนนี้มากกว่าฉัน |  |  |  |
| V5(a/b/c)_7: I am concerned about the cost of a COVID-19 vaccine | ฉันกังวลเกี่ยวกับค่าใช้จ่ายของวัคซีนโควิด-19 |  |  |  |
| V5(a/b/c)_8: It is against my religious beliefs | วัคซีนนี้ขัดแย้งกับความเชื่อทางศาสนาของฉัน |  |  |  |
| V5(a/b/c)_9: Other | อื่นๆ |  |  |  |
| V5(a/b/c)_10: ​​I don't trust the government | ฉันไม่ไว้วางใจรัฐบาล |  |  |  |
| V6: Why don’t you believe that you need a COVID-19 vaccine? Please select all that apply | เหตุใดคุณจึงไม่เชื่อว่าวัคซีนโควิด-19 จำเป็นสำหรับคุณ โปรดเลือกทุกข้อที่ใช่ | 1=Selected  0=Not selected -99 = missing/valid skipped/invalid -77 = seen but unanswered | ASK IF V5a_3/V5b_3/V5c_3 == 1 |  |
| V6_1: I already had COVID-19 | ฉันติดโควิด-19 ไปแล้ว |  |  |  |
| V6_2: I do not spend time with any high-risk people | ฉันไม่ได้ใช้เวลากับคนที่เสี่ยงติดไวรัสสูงเลย |  |  |  |
| V6_3: I am not a member of a high-risk group | ฉันไม่ใช่คนในกลุ่มเสี่ยงติดไวรัสสูง |  |  |  |
| V6_4: I plan to use masks or other precautions instead | ฉันวางแผนที่จะสวมหน้ากากหรือใช้วิธีการป้องกันอื่นๆ แทน |  |  |  |
| V6_5: I don't believe COVID-19 is a serious illness | ฉันไม่เชื่อว่าโควิด-19 เป็นโรคร้ายแรง |  |  |  |
| V6_6: I don't think vaccines are beneficial | ฉันไม่คิดว่าวัคซีนมีประโยชน์ |  |  |  |
| V6_7: Other | อื่นๆ |  |  |  |
| **MODULE A** |  |  |  |  |
| I6: How much do you trust the following sources to provide accurate news and information about COVID-19? | คุณเชื่อถือแหล่งข้อมูลดังต่อไปนี้มากน้อยเพียงใดในการให้ข่าวสารและข้อมูลที่ถูกต้องเกี่ยวกับโควิด-19 | 1=Do not trust  2=Somewhat trust  3=Trust  -99 = missing/valid skipped/invalid -77 = seen but unanswered  -88 = unseen because of module randomization | ASK IF module == A |  |
| I6_1: Local health workers, clinics, and community organizations | บุคลากรทางการแพทย์ คลินิก และองค์กรเพื่อชุมชนในพื้นที่ |  |  |  |
| I6_2: Scientists and other health experts | นักวิทยาศาสตร์และผู้เชี่ยวชาญด้านสุขภาพอื่นๆ |  |  |  |
| I6_3: World Health Organization (WHO) | องค์การอนามัยโลก (WHO) |  |  |  |
| I6_4: Government health authorities or officials | หน่วยงานหรือเจ้าหน้าที่ด้านสุขภาพของรัฐบาล |  |  |  |
| I6_5: Politicians | นักการเมือง |  |  |  |
| I6_6: Journalists | นักข่าว |  |  |  |
| I6_7: Friends and family | เพื่อนและครอบครัว |  |  |  |
| I6_8: Religious leaders | ผู้นำทางศาสนา |  |  |  |
| **MODULE B** |  |  |  |  |
| V10: Have you ever been told by a doctor, nurse, or other health professional that you have any of the following medical conditions? Please select all that apply. | แพทย์ พยาบาล หรือผู้เชี่ยวชาญด้านสาธารณสุขอื่นๆ เคยแจ้งคุณว่าคุณมีอาการเจ็บป่วยดังต่อไปนี้หรือไม่ โปรดเลือกทุกข้อที่ใช่ | 1=Selected  0=Not selected -99 = missing/valid skipped/invalid -77 = seen but unanswered  -88 = unseen because of module randomization | ASK IF module == B |  |
| V10_1: Asthma | โรคหอบหืด |  |  | Used with V10_2 to compute Chronic respiratory disease prevalence |
| V10_2: Chronic lung disease such as COPD, chronic bronchitis, or emphysema | โรคปอดเรื้อรัง เช่น COPD, โรคหลอดลมอักเสบชนิดเรื้อรัง หรือโรคถุงลมโป่งพอง |  |  | Used with V10_1 to compute Chronic respiratory disease prevalence |
| V10_3: Cancer | มะเร็ง |  |  |  |
| V10_4: Diabetes | เบาหวาน |  |  |  |
| V10_5: High blood pressure | ความดันโลหิตสูง |  |  |  |
| V10_6: Kidney disease | โรคไต |  |  |  |
| V10_7: Weakened or compromised immune system | ระบบภูมิคุ้มกันอ่อนแอ |  |  |  |
| V10_8: Heart attack, heart disease, or other heart condition | โรคหัวใจวาย โรคหัวใจ หรือสภาวะเกี่ยวกับหัวใจอื่นๆ |  |  |  |
| V10_9: Obesity | โรคอ้วน |  |  |  |
| V10_10: None of these | ไม่มีในตัวเลือกเหล่านี้ |  |  |  |
| **DEMOGRAPHICS** |  |  |  |  |
| E3: What is your gender? | โปรดระบุเพศของคุณ | 1=Male  2=Female  3=Other  4=Prefer not to answer -99 = missing/valid skipped/invalid -77 = seen but unanswered | ASK ALL |  |
| V11: Are you currently pregnant? | ขณะนี้คุณตั้งครรภ์อยู่หรือไม่ | 1 = Yes  2 = No -99 = missing/valid skipped/invalid -77 = seen but unanswered  -88 = unseen because of module randomization | ASK IF E3 != 1 AND module == B |  |
| E4: What is your age? | โปรดระบุอายุของคุณ | 1=18-24 years  2=25-34 years  3=35-44 years  4=45-54 years  5=55-64 years  6=65-74 years  7=75 years or older -99 = missing/valid skipped/invalid -77 = seen but unanswered | ASK ALL |  |
| **GEO VARS and PARADATA** |  |  |  |  |
| ISO_3: Country Code |  | Three character country code |  |  |
| NAME_1: Subregion Name |  | Name of province/subregion |  |  |

Full details available from the UMD-CTIS Microdata Repository in the codebook for Survey Version 11 (<https://covidmap.umd.edu/fbsurvey>).
