# Supplementary figures and images for: COVID-19 Vaccine Acceptance and Uptake in Bangkok, Thailand: Cross-sectional Online Survey
Source: JMIR Public Health Surveill. 2023 Apr 13;9:e40186. doi: 10.2196/40186 (PMC10141306; doi:10.2196/40186)

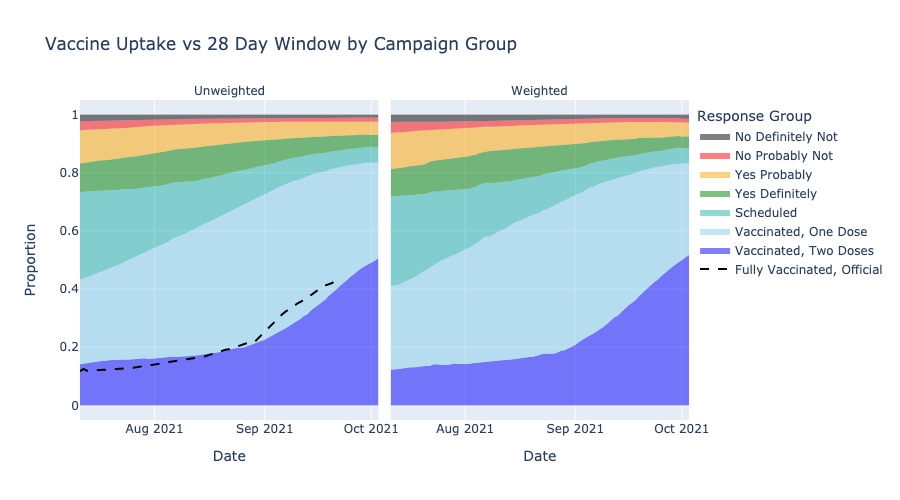

Supplement: Multimedia Appendix 2 [file publichealth_v9i1e40186_app2.png]

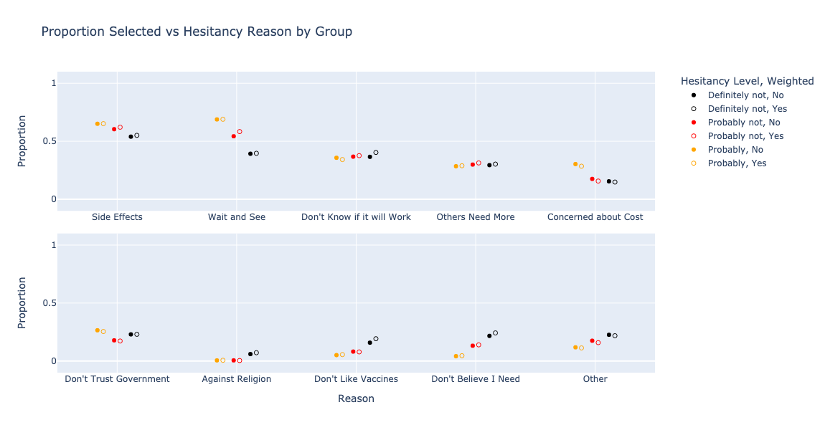

Supplement: Multimedia Appendix 3 [file publichealth_v9i1e40186_app3.png]

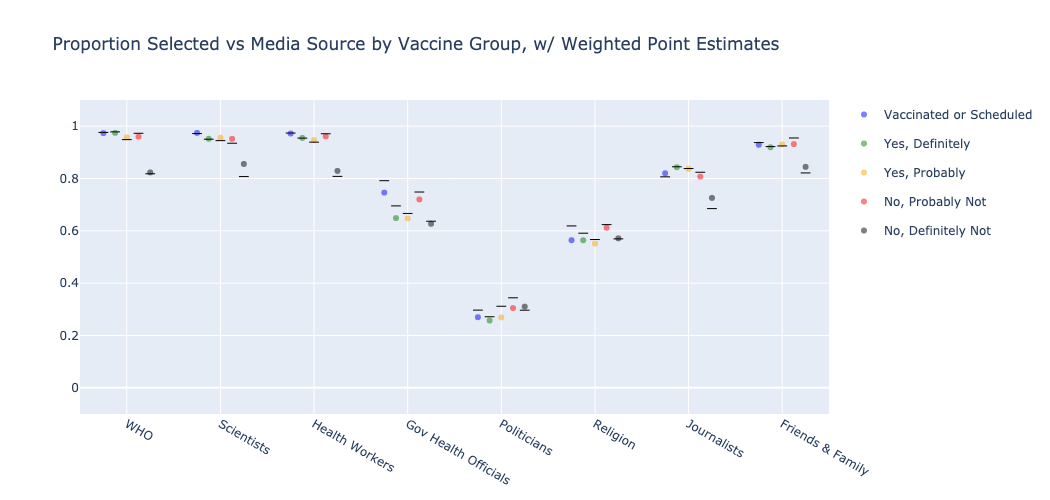

Supplement: Multimedia Appendix 4 [file publichealth_v9i1e40186_app4.png]

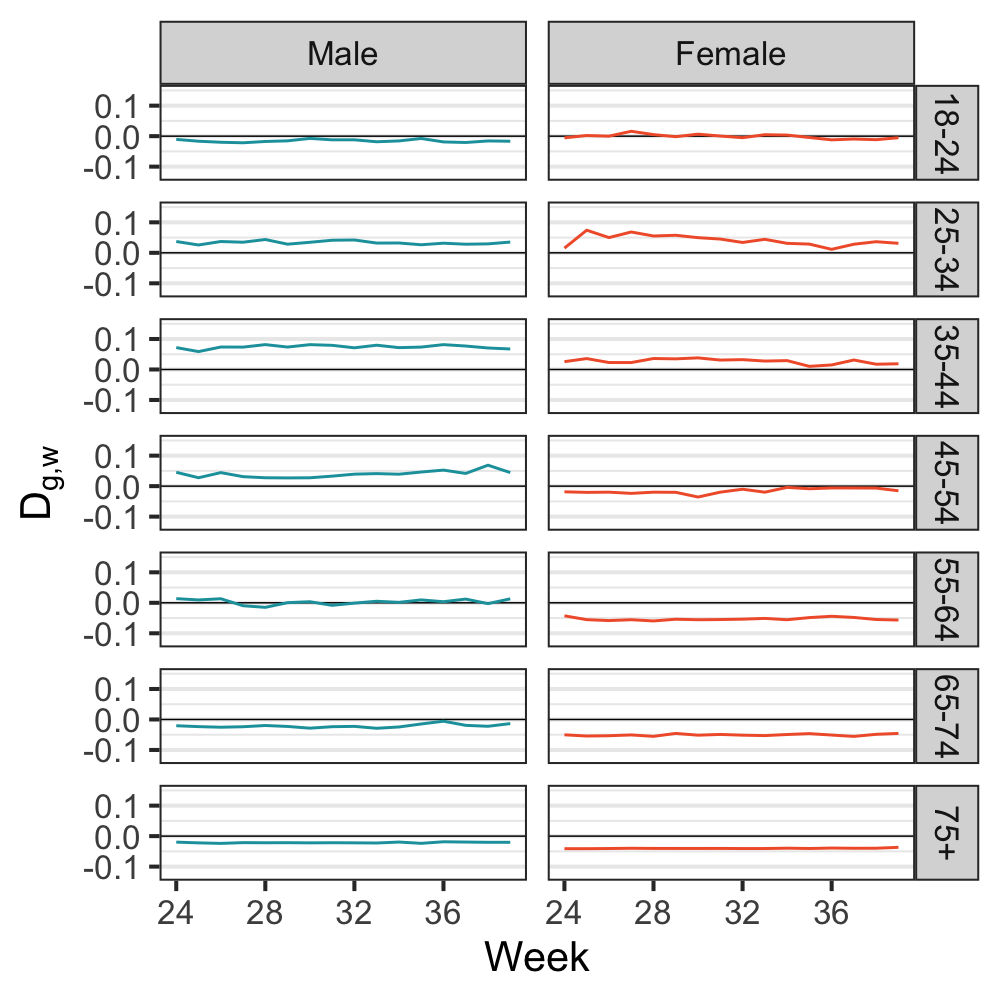

Supplement: Multimedia Appendix 6 [file publichealth_v9i1e40186_app6.png]

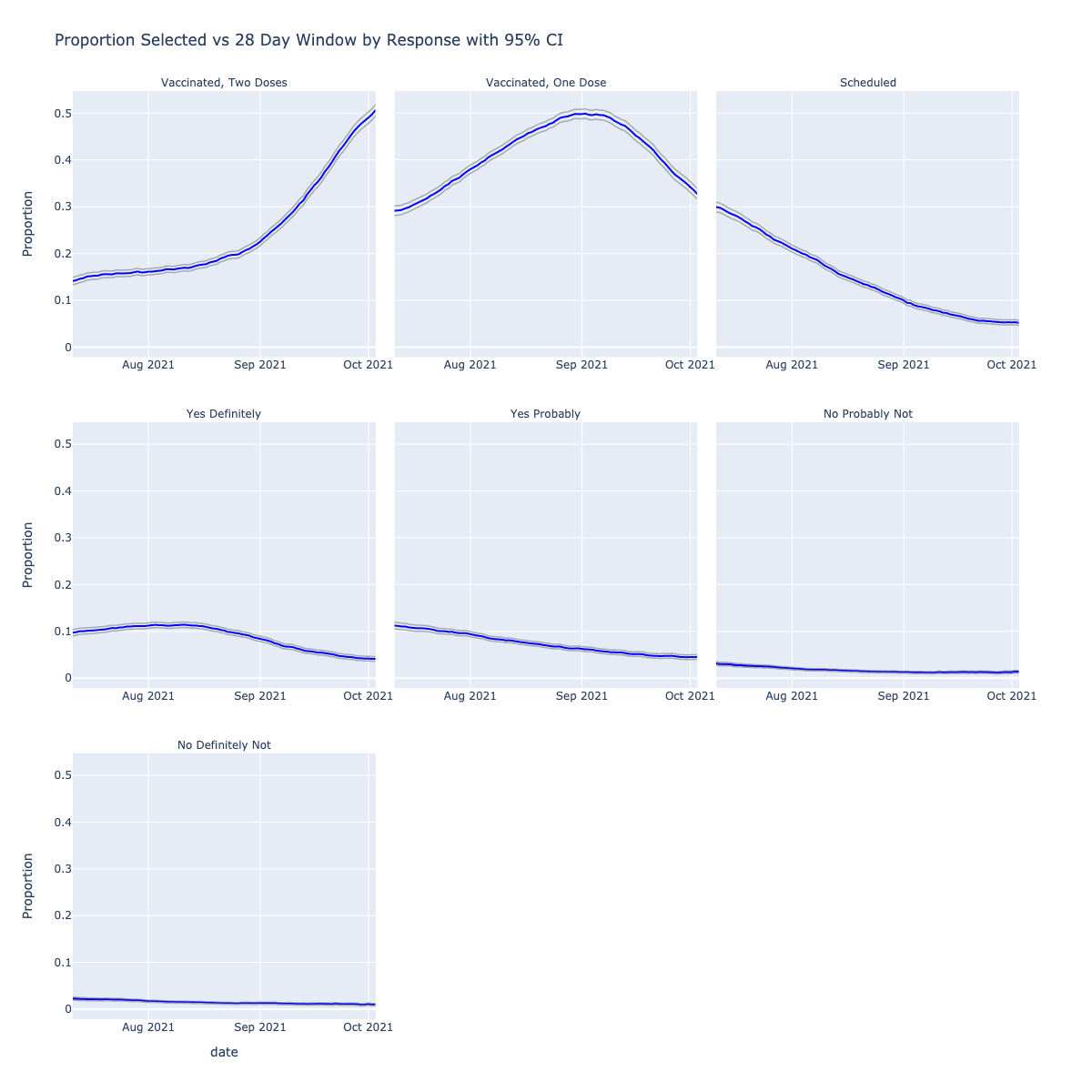

Supplement: Multimedia Appendix 8 [file publichealth_v9i1e40186_app8.png]

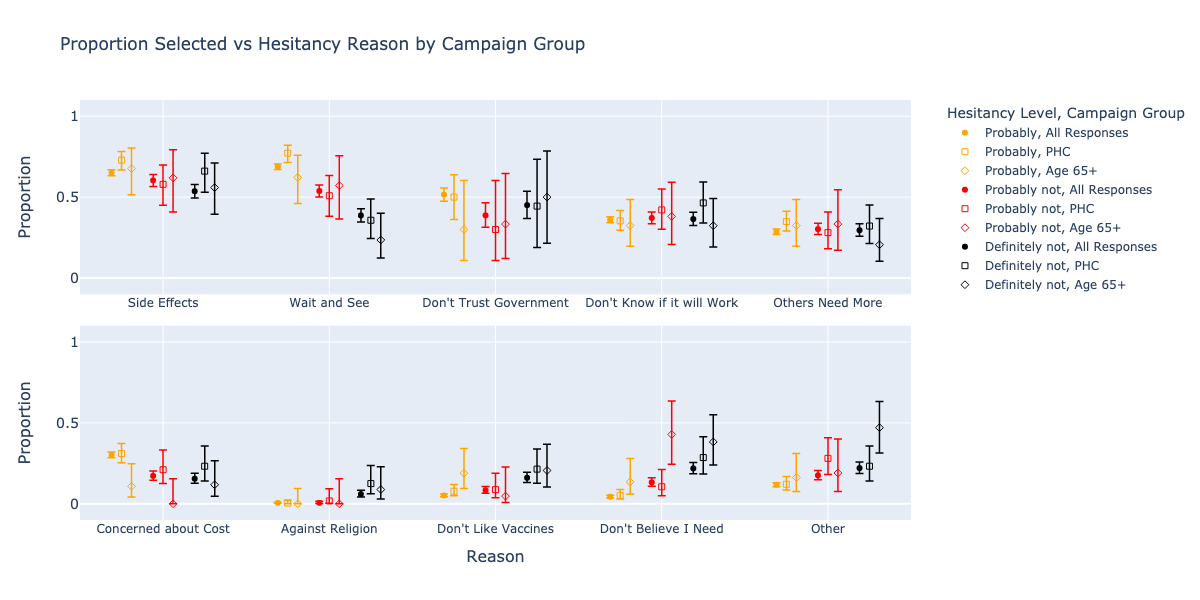

Supplement: Multimedia Appendix 9 [file publichealth_v9i1e40186_app9.png]

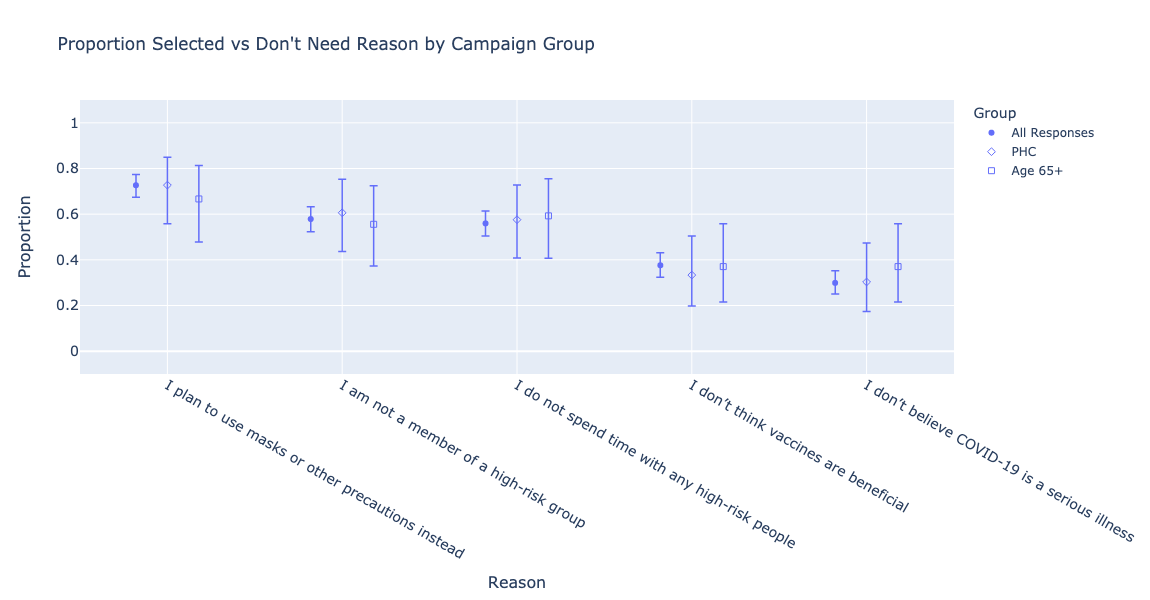

Supplement: Multimedia Appendix 10 [file publichealth_v9i1e40186_app10.png]
