# Supplementary material for: COVID-19 Vaccine Acceptance and Uptake in Bangkok, Thailand: Cross-sectional Online Survey
Source: JMIR Public Health Surveill. 2023 Apr 13;9:e40186. doi: 10.2196/40186 (PMC10141306; doi:10.2196/40186)
Supplement: Multimedia Appendix 5 [file publichealth_v9i1e40186_app5.docx]

**Multimedia Appendix 5.** Demographic and pre-existing health condition characteristics of survey respondents and that of the general population in Bangkok.

|  | | **n(%) Bangkok pop. age 18+ in UMD-CTIS**  **Total n=36,334** | **% Total Bangkok or Thailand pop. age 18+ in Census**  **Total n=4,479,801** |
| --- | --- | --- | --- |
|  |  |  |  |
| **Age-group** |  |  |  |
|  | 18-24 | 2759(7.6) | 10.4 |
|  | 25-34 | 7641(21.0) | 16.9 |
|  | 35-44 | 8907(24.5) | 18.9 |
|  | 45-54 | 6439(17.7) | 19.2 |
|  | 55-64 | 3569(9.8) | 17.0 |
|  | 65-74 | 1102(3.0) | 10.9 |
|  | 75+ | 163(0.4) | 6.7 |
|  | NA | 5754(15.8) | _ |
| **Sex** |  |  |  |
|  | Male | 16308(44.9) | 45.7 |
|  | Female | 13468(37.1) | 54.3 |
|  | Other | 331(0.9) | _ |
|  | Prefer not to answer | 477(1.3) | _ |
|  | NA | 5750(15.8) | _ |
| **PHC ^+^** | |  |  |
|  | **At least one condition** | **3407(17.3)** | _ |
|  | Obesity | 1555(7.9) | _ |
|  | Diabetes | 1090(5.5) | 8.4 |
|  | Chronic respiratory diseases ^~^ | 641(3.2) | 6.2 |
|  | Asthma | 575(2.9) | _ |
|  | Cardiovascular diseases | 488(2.5) | 9 |
|  | Chronic kidney disease | 207(1.0) | 19.6 |
|  | Cancer | 171(0.9) | 6.9 |
|  | Chronic lung diseases | 155(0.8) | _ |
|  | Pregnant ^ | 92(0.3) | _ |
| **Vaccine uptake and hesitancy** | |  |  |
|  | Vaccinated, two doses | 8596(23.7) | _ |
|  | Vaccinated, one dose unspecified dose | 13712(37.7) | _ |
|  | Scheduled | 5505(15.2) | _ |
|  | Definitely willing | 2909(8.0) | _ |
|  | Probably willing | 2619(7.2) | _ |
|  | Probably not willing | 652(1.8) | _ |
|  | Definitely not willing | 522(1.4) | _ |
| The demographic data of the general population was obtained from the 2021 Thailand census and the PHC estimation data was obtained from the 2019 Global Burden of Disease Study.  **^+^** 16600(45.7%) did not receive the module about PHC  ^~^ includes UMD-CTIS responders who self identified to be diagnosed for Asthma or Chronic lung diseases such as COPD, chronic bronchitis, or emphysema | | | |
| ^ 7405(25.0%) responded No | |  |  |
