# Supplementary material for: COVID-19 Vaccine Acceptance and Uptake in Bangkok, Thailand: Cross-sectional Online Survey
Source: JMIR Public Health Surveill. 2023 Apr 13;9:e40186. doi: 10.2196/40186 (PMC10141306; doi:10.2196/40186)
Supplement: Multimedia Appendix 7 [file publichealth_v9i1e40186_app7.docx]

**Multimedia Appendix 7.** Results for simple linear regression for week versus difference in the proportion of each of the 14 age-gender groups in University of Maryland COVID-19 Trends and Impact Survey versus Bangkok census demographics.

| **Gender** | **Age** | **Intercept** | **Slope** | ***P*** |
| --- | --- | --- | --- | --- |
| Female | 18-24 | 0.02590404 | -0.0008546 | .03 |
| Female | 25-34 | 0.10386954 | -0.0019866 | .03 |
| Female | 35-44 | 0.05279347 | -0.0008274 | .06 |
| Female | 45-54 | -0.0510414 | 0.0011159 | .01 |
| Female | 55-64 | -0.0574733 | 0.00014197 | .60 |
| Female | 65-74 | -0.0577432 | 0.00022221 | .20 |
| Female | 75+ | -0.0448505 | 0.00015166 | .004 |
| Male | 18-24 | -0.0141277 | -0.00000414 | .87 |
| Male | 25-34 | 0.04360287 | -0.0003136 | .31 |
| Male | 35-44 | 0.06883707 | 0.00016678 | .63 |
| Male | 45-54 | -0.0030483 | 0.00136004 | .02 |
| Male | 55-64 | -0.002825 | 0.00018637 | .71 |
| Male | 65-74 | -0.0387316 | 0.00055451 | .08 |
| Male | 75+ | -0.0251646 | 0.00012401 | .14 |
